# Using Smart Technology to Improve Outcomes in Myocardial Infarction Patients: Rationale and Design of a Protocol for a Randomized Controlled Trial, The Box

**DOI:** 10.2196/resprot.8038

**Published:** 2017-09-22

**Authors:** Roderick Willem Treskes, Louise Anna van Winden, Nicole van Keulen, Douwe Ekke Atsma, Enno Tjeerd van der Velde, Elske van den Akker-van Marle, Bart Mertens, Martin Jan Schalij

**Affiliations:** ^1^ Department of Cardiology Leiden University Medical Center Leiden Netherlands; ^2^ Department of Medical Decision Making Leiden University Medical Center Leiden Netherlands; ^3^ Department of Medical Statistics Leiden University Medical Center Leiden Netherlands

**Keywords:** telemedicine, myocardial infarction, hypertension

## Abstract

**Background:**

Recent evidence suggests that frequent monitoring using smartphone-compatible wearable technologies might improve clinical effectiveness and patient satisfaction of care.

**Objective:**

The aim of this study is to investigate the clinical effectiveness and patient satisfaction of a smart technology intervention in patients admitted with a ST elevation myocardial infarction (STEMI) or non-ST acute coronary syndrome (NST-ACS).

**Methods:**

In this single center, open, randomized controlled trial patients who suffered from STEMI or NST-ACS will be randomized 1:1 to an intervention group or control group. Both groups will be followed up to one year after the index event. The intervention group will take daily measurements with a smartphone-compatible electrocardiogram device, blood pressure (BP) monitor, weight scale, and activity tracker. Furthermore, two of four outpatient clinic visits will be replaced by electronic visits (1 and 6 months after index event). The control group will receive regular care, consisting of four outpatient clinic visits (1, 3, 6, and 12 months after index event). All patients will be asked to fill in validated questionnaires about patient satisfaction, quality of life, propensity of medication adherence, and physical activity.

**Results:**

The primary outcome of this trial will be percentage of patients with controlled BP. Secondary outcomes include patient satisfaction, health care utilization, major adverse cardiac events, medication adherence, physical activity, quality of life, and percentage of patients in which a sustained arrhythmia is detected.

**Conclusions:**

Smart technology could potentially improve care in postmyocardial infarction patients. This trial will investigate whether usage of smart technology can improve clinical- and cost-effectiveness of care.

**Trial Registration:**

Clinicaltrials.gov NCT02976376; https://clinicaltrials.gov/ct2/show/NCT02976376 (Archived by WebCite at http://www.webcitation.org/6tcvAdbdH)

## Introduction

Current European Society of Cardiology guidelines on secondary prevention in patients with sustained ST elevation myocardial infarction (STEMI) or acute coronary syndrome without persistent ST elevation (non-ST acute coronary syndrome; NST-ACS) recommend tight blood pressure (BP) control, weight control, and adequate physical activity after discharge from the hospital, as well as regular electrocardiograms (ECGs) [1,2]. In current practice, these patients regularly visit outpatient clinics in the first year after their initial hospitalization, where the patient is interviewed, weighed, an ECG is recorded, BP is measured, lifestyle advices are given, and pharmaceutical treatment is evaluated [3]. In this situation, patients must be physically present at the outpatient clinic [3], which might pose a burden to the patient, especially in remote areas [4]. Furthermore, this process necessitates trained health care staff, which increases their workload.

Recent advances in information and communication technologies have enabled remote monitoring of vital signs and remote doctor-patient contacts (together encompassing part of the broad concept of *telemedicine*) [5-9]. In recent years, a number of smartphone-compatible wearables have received a European Conformity (CE)-mark and Food and Drug Administration (FDA) clearance, and are available for over-the-counter sale in the European Union and the United States [10]. Some of these smartphone-compatible wearables allow for the measurement of (depending on the type of wearable) the number of steps taken per day, BP, weight, and the recording of an ECG. The devices are easy-to-use and do not require the assistance of trained health care staff. Results of measurements are communicated with smartphone apps tailored to the specific device. Data is uploaded via the Internet to servers belonging to the manufacturers of the devices [10,11].

Recent research in various patient populations suggests that telemedicine might improve clinical effectiveness and patient satisfaction of care [12,13]. Remote and more frequent monitoring with subsequent therapy changes has been shown to improve clinical outcomes of patients with uncontrolled hypertension (achieving 18.4% more patients with controlled BP) [12] and with type 2 diabetes mellitus (a statistically significant 0.37% reduction in hemoglobin A1c) [13]. Furthermore, remote video contact moments, in which the doctor and patient communicate via a video connection, are potentially time-saving for patients [14,15]. One study found that office visits required an average of 50 minutes of a patient’s time, while electronic visits only required 22 minutes on average [15].

We therefore hypothesize that telemedicine improves clinical effectiveness and patient satisfaction of care in the follow-up of STEMI and NST-ACS patients. Thus, the aim of this study is to investigate the clinical effectiveness and patient satisfaction of a smart technology intervention in patients after being admitted with a STEMI or NST-ACS. In this paper, the rationale and design of this open, single center, randomized controlled trial (RCT) are presented.

## Methods

### Study Design (Design, Randomization, and Follow-Up)

*The Box* is a single-center open RCT, and is a parallel group study. The study will be conducted at the Leiden University Medical Center (LUMC), a tertiary care hospital in Leiden, The Netherlands. The trial is registered under clinical trial number NCT02976376 (Clinicaltrials.gov) and NL56453.058.16 (Toetsingonline.nl). After inclusion, patients will be randomized 1:1 to either *The Box* (intervention group) or to regular follow-up (control group). Block randomization per 10 participants will be performed. Randomization will be stratified per primary diagnosis (STEMI or NST-ACS) and per age (<50, 51-60, 61-70, 71-80, and >80 years). A website will be used to generate randomization lists [16].

### Patient Population

Patients who are admitted to the cardiology department of the LUMC with STEMI [1] or NST-ACS [2] will be eligible for participation. Patients with a STEMI or NST-ACS who match the inclusion and exclusion criteria will be approached for participation in the protocol within 24 hours after primary percutaneous coronary intervention (PCI). The maximum time between primary PCI and study inclusion is 96 hours. All inclusion and exclusion criteria are listed in Textbox 1.

**Inclusion and exclusion criteria.**

Inclusion criteria

- Patient is admitted with acute myocardial infarction
- Patient is able to communicate in English or Dutch

Exclusion criteria

- Body Mass Index >35 kg/m^2^
- Included in another RCT
- Does not have wireless Internet access at home
- Less than 18 years old
- Considered an incapacitated adult (this decision is left to the discretion of the responsible cardiologist)
- Pregnant
- Unwilling to sign the informed consent form

### Regular Follow-Up

Since 2004, the department of Cardiology of the LUMC has a dedicated care track for patients with STEMI or NST-ACS. Details about this protocol have been described previously by Liem et al [3]. Briefly, patients with signs and symptoms that are possibly related to a myocardial infarction are referred to a PCI center. Upon arrival, patients are immediately transferred to the catheterization department, where primary PCI of the culprit lesion is performed. Before discharge, patients are given written and oral information on the importance of medication adherence and lifestyle advices, in accordance with the European Guidelines on cardiovascular disease prevention in clinical practice [17].

After approximately 48-hours, patients are discharged from the hospital. The standard follow-up during the first year after discharge includes four outpatient clinic visits:

1. *Approximately 1 month after STEMI or NST-ACS*. This visit includes: a BP measurement; a 10-second, 12-lead ECG; laboratory testing (including kidney function, renal function, and lipid spectrum); and an interview with a doctor or nurse practitioner.
2. *Approximately 3 months after STEMI or NST-ACS*. This visit includes: a BP measurement; a 10-second, 12-lead ECG; stress echo; a 24-hour Holter ECG, and an interview with a doctor or nurse practitioner.
3. *Approximately 6 months after STEMI or NST-ACS*. This visit includes: a BP measurement, a 10-second, 12-lead ECG; a 24-hour Holter ECG; laboratory testing (including kidney function, renal function, and lipid spectrum); a transthoracic echocardiogram (TTE); and an interview with a doctor or nurse practitioner.
4. *Approximately 12 months after STEMI or NST-ACS*. This visit includes: a BP measurement, a 10-second, 12-lead ECG; laboratory testing (including kidney function, renal function, and lipid spectrum); a TTE; and an interview with a doctor or nurse practitioner.

**Figure 1:**
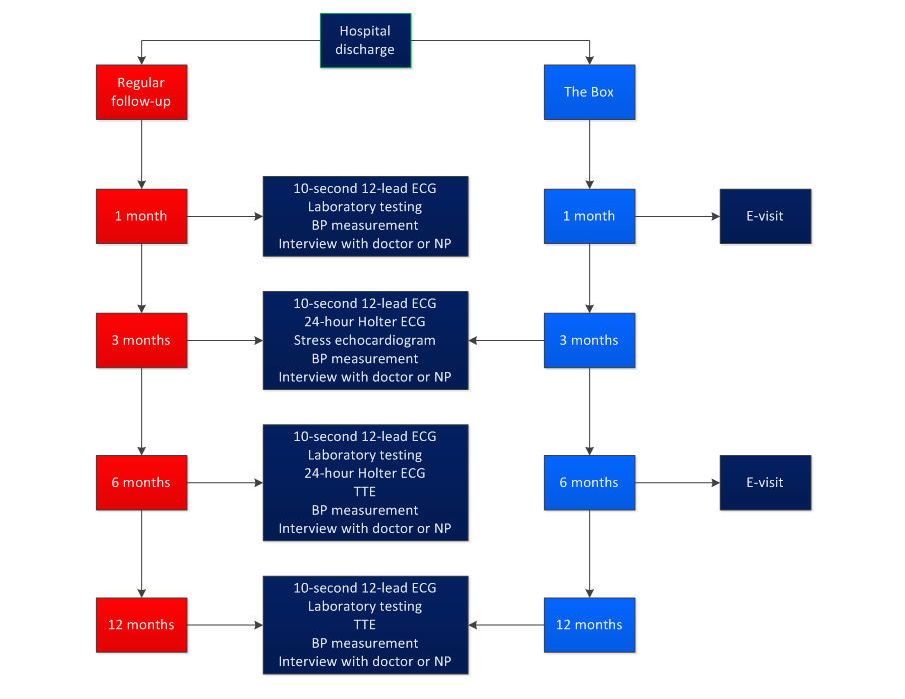
MISSION, follow-up of patients who suffered from STEMI or NST-ACS. BP: blood pressure; ECG: electrocardiogram; NP: nurse practitioner; TTE: transthoracic echocardiogram.

Patients who are randomized to regular follow-up receive the same care as patients who do not participate in the study. A flowchart of regular follow-up is detailed in Figure 1.

**Figure 2:**
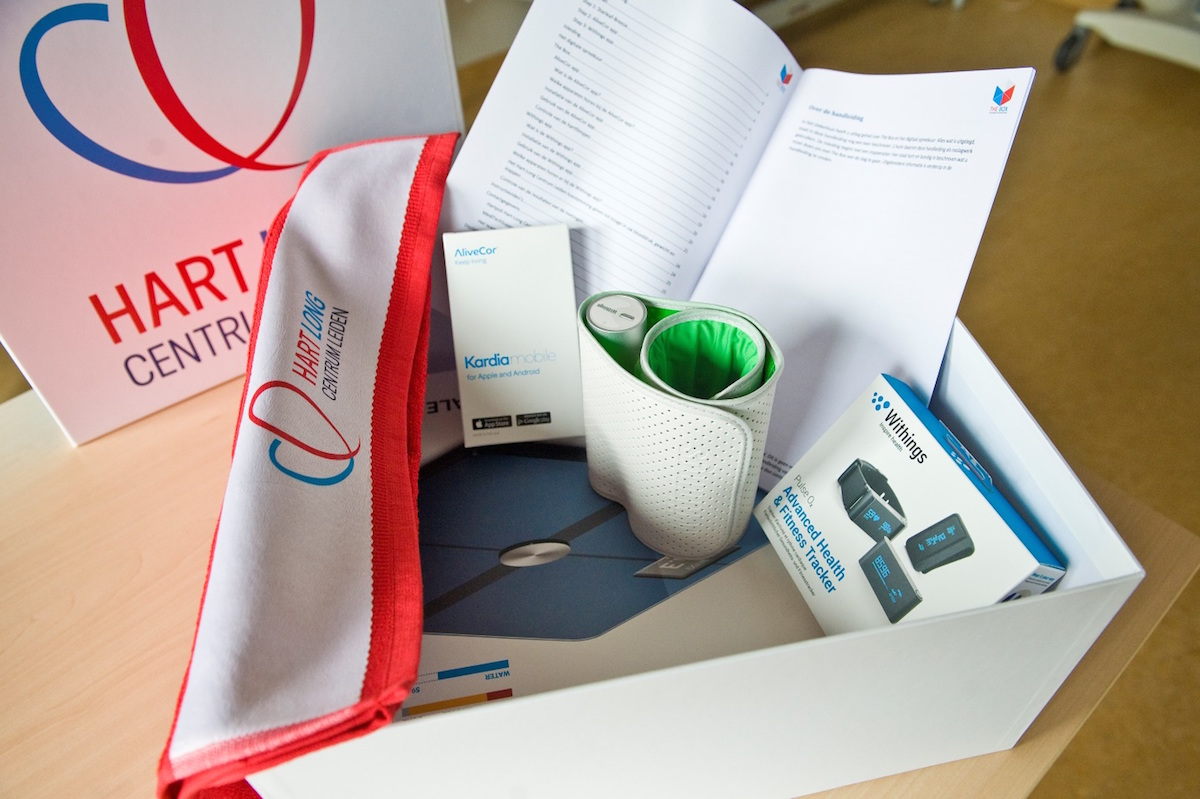
The Box.

### The Box

When randomized to *The Box* (Figure 2), patients will receive a box containing a weight scale, BP monitor, activity tracker, and a wearable ECG device. Patients will receive *The Box* before discharge and will be given the same written and oral information on the importance of medication adherence and lifestyle advices as the control group, in accordance with the European Guidelines on cardiovascular disease prevention in clinical practice [17]. The necessary apps will be downloaded on the patient’s smartphone; necessary accounts will be created and the installation of the devices will be carried out by a health care professional dedicated to the project. Afterwards, patients will be given oral instructions on the usage of the devices: they will be instructed to measure BP in a sitting position after five minutes of resting. The device should be applied to the left upper arm. Patients will be instructed to rest their left forearm on a table. Furthermore, a manual explaining the usage of the wearables (described below) will be handed over with *The Box*. Instruction videos are also available on YouTube. Patients who do not own a smartphone or tablet with iOS or Android OS but are willing to participate and be randomized to *The Box* will receive a smartphone. Patients will be instructed to use their own wireless Internet access (eg, home WiFi network). No mobile data network plan will be provided with the smartphone.

Patients will be instructed to record a single lead ECG, measure BP, and record their weight daily, preferably at the same time of day. Furthermore, participants will be asked to record a single lead ECG if any symptoms of possible cardiac origin occur (as interpreted by the patient). Lastly, patients will be instructed to wear their activity tracker during the day to track their daily number of steps, and at night to track the duration and quality of sleep. Patients will be told that measurements will be checked on a daily basis and that they will be contacted in cases of predefined data irregularities. Patients will be explicitly told that they cannot rely on the devices of *The Box* in emergency situations.

In addition to daily measurements, the first and third of the four standard outpatient clinic visits will be replaced by an electronic visit, in which the patient will communicate with the doctor or nurse practitioner via a secured video connection. The content of the interview will be comparable to the content of a regular outpatient clinic visit. The same doctors and nurse practitioners will perform the regular outpatient clinic visits and the digital outpatient clinic visits. In the intervention group, the 10-second 12-lead ECG and the laboratory testing one month after the index event will not be performed. Moreover, the 10-second 12-lead ECG, the 24-hour Holter ECG, laboratory testing, and the TTE 6 months after the index event will not be performed (Figure 1).

### Devices

All devices used for this study are noninvasive, battery powered, smartphone-compatible devices. All devices have a CE-mark, are approved by the United States FDA, and are allowed for over-the-counter sale in the European Union and the United States. The installation and usage of the devices are so intuitive that no medical staff needs to assist when the devices are used by the patient.

The usage of the devices requires a smartphone or tablet with Android Operating System (OS; Google, Mountain View, California, USA) or iOS (Apple Computers, Cupertino, CA, USA). The devices communicate with a dedicated mobile app on the smartphone or tablet, which can be downloaded from the App Store (iOS) or Play Store (Android). The data from the measurements are stored on the smartphone or tablet and uploaded to the app manufacturer’s servers (the cloud), which are located in Europe. An Internet connection (eg, WiFi, 3G, or 4G) is required to synchronize with the cloud. Measurements can be done while the smartphone or tablet is offline. In these cases, the results of the measurements are stored on the smartphone or tablet, and uploaded to the server when the smartphone is reconnected to the Internet.

#### Electrocardiogram Device

The ECG device (AliveCor, AliveCor Inc., San Francisco, CA, USA) contains two electrodes. The device communicates with the AliveCor app. The ECG device allows the user to record a 30-second single lead ECG. To record an ECG, the patient must position two or three fingers of the right hand on one electrode and two or three fingers of the left hand on the other electrode. The device is to be held within approximately 1 to 30 centimeters of the smartphone. An ultrasound signal is sent from the ECG device to the smartphone. This signal is then converted to a live single lead ECG that is subsequently shown on the smartphone screen [7,10].

After 30 seconds, an automated algorithm in the app calculates the R-R intervals and formulates a diagnosis, varying from *normal* to *possible atrial fibrillation* to *undetermined*, on the screen [10]. The patient then has the ability to add notes, and is requested to report any symptoms (if present) before saving the ECG.

#### Blood Pressure Monitor

The BP monitor (Withings S.A., Issy les Moulineaux, France) is a smartphone-compatible, battery operated oscillometric BP cuff. The device allows the user to measure systolic BP, diastolic BP, and heart rate. The device is applied around the left or right upper arm (depending on the patient’s preference). Upon pushing the button on the cuff, a connection is made with the smartphone via Bluetooth. The inflation and deflation of the cuff is automated and can be initiated via the dedicated Withings Health Mate app (for iOS and Android) on the smartphone. The average duration of a measurement is approximately 20 seconds. After inflation and deflation, the systolic blood pressure (SBP), diastolic blood pressure (DBP), and heart rate are shown on the smartphone screen.

#### The Weight Scale

The weight scale (Withings S.A., Issy les Moulineaux, France) allows the patient to track weight, fat percentage, heart rate, and ambient CO_2_ parts per million. To measure all four parameters, the patient must stand on the weight scale. While standing on the weight scale, the patient must select their own account. The results are shown on a screen on the weight scale, and are automatically uploaded via the Internet to the Withings server.

#### Activity Tracker

The activity tracker (Pulse Ox, Withings S.A., Issy les Moulineaux, France) allows the patient to track the number of steps taken per day. The device is the size of a thumb and can be attached to the wrist or belt, and also allows the patient to track duration and quality of sleep. Steps are automatically tracked. The measurement results are sent via Bluetooth to a dedicated smartphone app, which is compatible with iOS and Android OS.

### Storage of the ECGs

**Figure 3:**
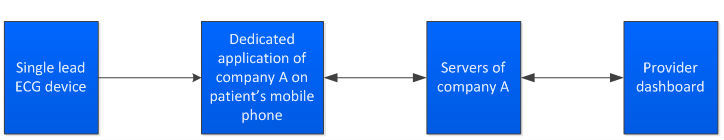
Data integration of single lead electrocardiograms. Company A is the ECG manufacturer. ECG: electrocardiogram.

**Figure 4:**
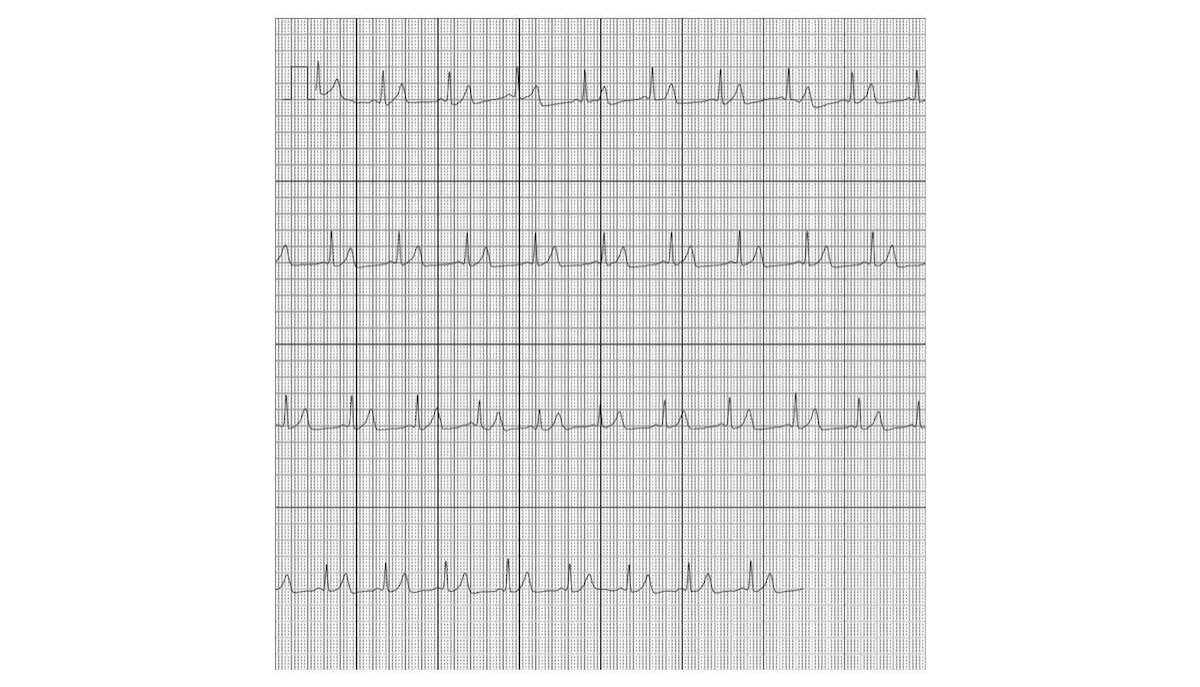
A PDF generated by the ECG device, showing sinus rhythm.

**Figure 5:**
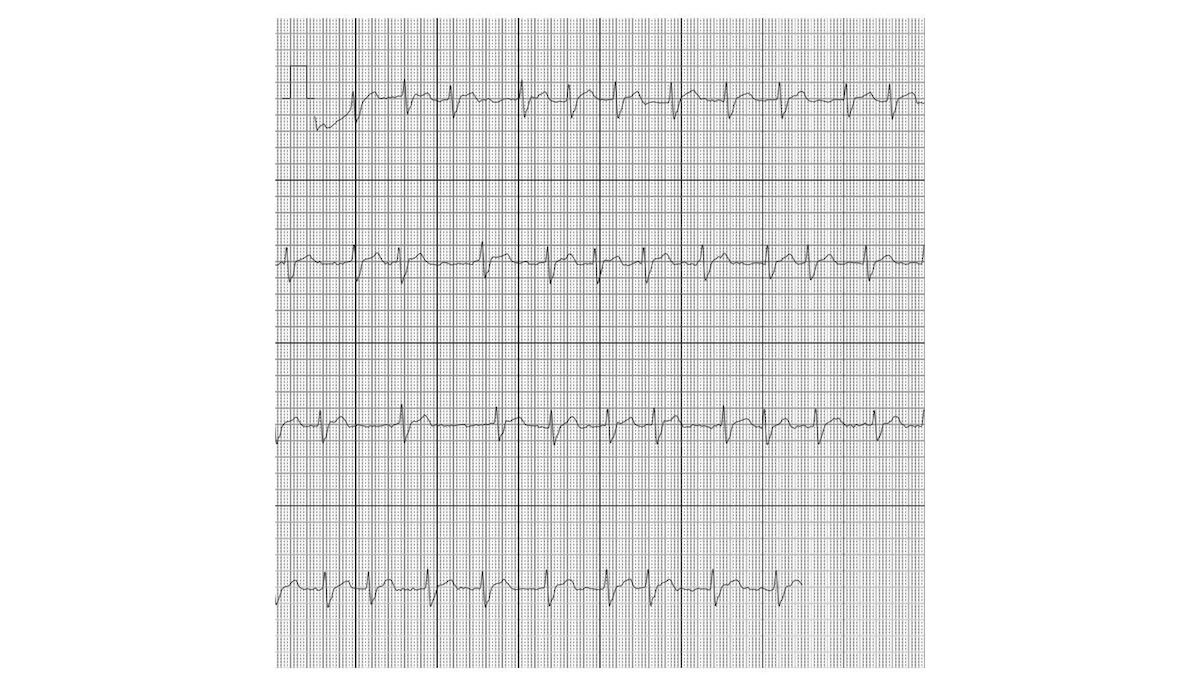
A PDF, generated by the ECG device, showing atrial fibrillation.

Single lead ECGs, generated by the single lead ECG device, are stored in the cloud. The system offers patients the ability to connect their personal account with a physician’s account. The physician can then review the ECGs made by patients linked to their account, including the diagnosis given by the app’s algorithm and the symptoms reported by the patient. The automated diagnosis algorithm has a reported sensitivity of 97% and a specificity of 98% for the detection of atrial fibrillation [7]. ECGs that are classified by AliveCor as *possible atrial fibrillation* and *undetermined* will subsequently be checked by a project-dedicated health care professional in our department. A patient will be contacted if a previously undiagnosed arrhythmia is seen or if a patient repeatedly reports symptoms. A flowchart of the storage of the ECGs is shown in Figure 3. An example of a portable document format (PDF) ECG showing sinus rhythm generated by the ECG device is shown in Figure 4. An example of a PDF ECG showing atrial fibrillation generated by the ECG device is shown in Figure 5.

### Data Integration in Electronic Medical Records

**Figure 6:**
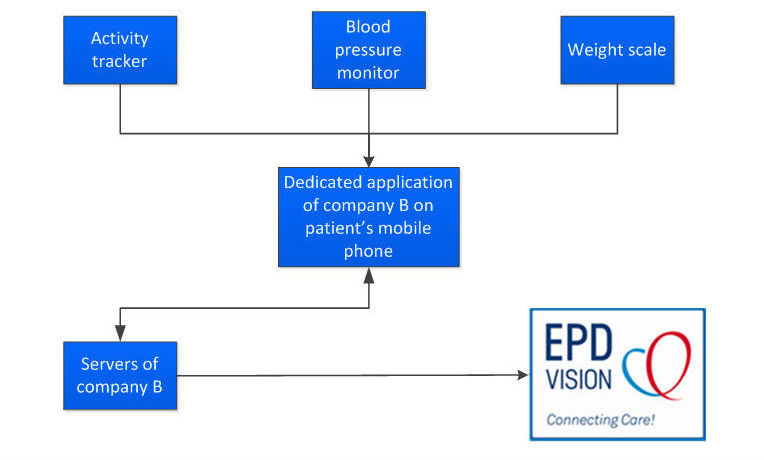
Data integration of the activity tracker, weight scale, and BP monitor in the department’s Cardiology Information System “EPD-Vision”. Company B is the manufacturer of the activity tracker, blood pressure monitor, and weight scale.

The measurement results from the weight scale, BP monitor, and activity tracker will be stored on the manufacturer’s server (Withings S.A., Issy les Moulineaux, France). Data will be extracted from the Withings server and integrated in the department’s dedicated electronic medical record (EMR; EPD-Vision, Department of Cardiology, LUMC, The Netherlands). An automated algorithm searches for predefined irregularities in the data (Textbox 2). Data can subsequently be displayed in graphic format to facilitate trend analysis. A flowchart of the storage of BP, weight, and activity data is displayed in Figure 6. If bugs arise in this system, a software developer who works at the Department of Cardiology will fix the bug.

**Warnings generated by the dedicated system.**

BP monitor

- If a patient has not sent BP results for more than 7 consecutive days
- If the heart rate is >100 beats per minute
- If the SBP is >139
- If the DBP is > 89
- If the SBP is 10 mmHg higher than last measurement
- If the DBP is 5 mmHg higher than last measurement
- If the SBP is 10 mmHg higher than 7 measurements before
- If the DBP is 5 mmHg higher than 7 measurements before
- If the SBP is 10 mmHg lower than last measurement
- If the DBP is 5 mmHg lower than last measurement
- If the SBP is 10 mmHg lower than 7 measurements before
- If the DBP is 5 mmHg lower than 7 measurements before

Weight scale

- If a patient has not sent measurements for more than 7 consecutive days
- If the weight is more than 2 kg higher than last measurement
- If the weight is more than 3 kg higher than 7 measurements before
- If the weight is more than 2 kg lower than last measurement
- If the weight is more than 3 kg lower than 7 measurements before

Activity tracker

- If a patient has not sent measurements for more than 7 consecutive days

### Reasons to Contact Patients

Data will be checked daily by a project-dedicated health care professional. When prespecified irregularities are seen in the data, a patient will be contacted within 48 hours by email or phone. These data irregularities are standardized and shown in Textbox 2.

### Reasons to Adjust Therapeutic Regimen

Data will be discussed by the data reviewer and the patient’s treating physician or nurse practitioner. There are several reasons to contact the patient, which are detailed below.

#### BP Monitor

Warnings generated by the system on the basis of BP measurements will be reviewed by a project-dedicated health care professional. The reason to change medication will be left to the discretion of the treating physician.

#### Single Lead Electrocardiogram

In cases of newly diagnosed arrhythmias lasting at least 30 seconds (eg, atrial fibrillation, atrial flutter, nodal or ventricular escape rhythms, ventricular tachycardias) or at least 4 newly diagnosed asymptomatic premature ventricular contractions, a 24-Hour Holter ECG will be performed. Patients noting chest pain or shortness of breath as symptoms will be contacted for an interview by telephone. The decision to change medication or to refer the patient for invasive therapy will be left to the discretion of the treating physician.

#### Weight

Weight will not be a primary reason to change a therapeutic regimen. Patients can be given lifestyle advice, depending on their height, weight, and estimated fat percentage; this will only be done at scheduled outpatient clinic visits.

#### Activity

Activity tracking data is not a primary reason to change a therapeutic regimen. Patients can be advised to exercise more or less, depending on the data; this will only be done at scheduled outpatient clinic visits.

### Nonadherence

If a patient does not send measurements from any of their four devices for 21 consecutive days, they will be considered nonadherent. A standardized email will be sent to the patient, stating that measurements have not been received and that they are urged to contact the hospital in case of any technical difficulties. If no answer is received within 21 days or no measurements are seen within 21 days, the patient will be called by telephone. Any technical difficulties or objections by the patient will be assessed and solved if possible. After this phone call, if the patient starts sending measurements, they will be considered adherent again. This patient will be sent a standardized email in case they become nonadherent again. If the patient does not start sending measurements, they will not be approached again by email or by telephone to try to affect their nonadherence. However, this patient will not be excluded from the trial, and will still be followed-up according to *The Box* protocol. If patients notify the hospital that they want to have regular outpatient clinic visits, they will be followed-up according to the regular follow-up protocol.

### Questionnaires

All patients, in both the intervention and control groups, will be asked to fill-in a short form health questionnaire (SF-36) [18], a patient satisfaction questionnaire [19], a medication adherence questionnaire, and an International Physical Activity Questionnaire (iPAQ; to assess the patient’s level of physical activity) [20]. These questionnaires will be used within one month after myocardial infarction, six months after myocardial infarction, and twelve months after myocardial infarction.

### Privacy of Study Participants

To anonymize the data, patients will receive an email address consisting of a study code, which they can use to create their Withings and AliveCor accounts. The corresponding names will be kept in a separate, password-protected database.

### Ethical Conduct

The study is approved by the Hospital’s Medical Ethics Committee (P16.070). All procedures will be conducted in accordance with the principles of the Declaration of Helsinki (version 10, October 2013) and in accordance with the Dutch Medical Research Involving Human Subjects Act (WMO) and Good Clinical Practice. Written offline informed consent will be obtained from all participants. The devices used in this study and described above have been approved by our Hospital’s Instrumentation Department. All devices are CE-marked and are available for sale in the European Union. All devices have been purchased by our department for this study. No manufacturer has a role in the study design, data collection, statistical analysis, or writing of the manuscript. No financial support was received for this study from any device manufacturer. All devices are provided to study participants who are randomized to *The Box* group free of charge.

### Study Withdrawal

Patients who are randomized to *The Box* group can be withdrawn from the study if they are either nonadherent (as discussed above) or if they themselves wish to withdraw from the study. Patients who withdraw from regular follow-up are considered lost to follow-up.

## Results

### Study Outcomes

The primary outcome of this study is the percentage of patients with normalized BP (defined as SBP <140 mmHg and DBP <90 mmHg), as measured at the 12-month outpatient clinic visit. Secondary outcomes of this study are detailed in Textbox 3.

**Secondary outcome measures.**

1. The percentage of patients with controlled BP (defined as SBP <140 mmHg and DBP <90 mmHg), as measured at the 3-month outpatient clinic visit.
2. Patient satisfaction, as assessed by the patient satisfaction questionnaire [18].
3. Health care utilization, defined as an outpatient clinic visit, emergency care visit, or admission for any reason. This factor will be measured via questionnaires and verified by EMR data.
4. Major Adverse Cardiac Events (MACEs)
  a. Death of any cause
  b. Cardiac death
  c. Recurrent STEMI
  d. Recurrent NST-ACS
  e. Revascularization
  f. Hospitalization for heart failure
  g. Transient Ischemic Attack
  h. Ischemic stroke
5. Propensity of medication adherence, measured by the 8-item Morisky Medication Adherence Scale (MMAS-8) [21].
6. Physical activity, as measured by the iPAQ [20].
7. Quality of life, measured by the SF-36 [19].
8. Percentage of patients in which a previously unknown sustained arrhythmia (>30 seconds) is detected.
9. Cost-effectiveness, expressed as the incremental cost-effectiveness ratio.

### Economic Analysis

To assess the cost-effectiveness of the intervention, costs per quality adjusted life year (QALY) will be calculated. The analysis will be performed from a societal perspective with a time horizon of one year. Patients will receive a health care resource use questionnaire at 6 months and 12 months. In this questionnaire, a patient will be asked to fill in their total health care utilization, such as outpatient clinic visits, emergency visits, admissions for any reason, and visits to the general practitioner. All outpatient clinic visits, emergency visits, and admissions for any reason reported in the questionnaire will be verified by EMR data. In the same questionnaire, the patient will be asked to fill in total medication use. All health care and medication use will be multiplied with standard cost prices to calculate costs [22]. To calculate indirect costs, patients will be asked to note absence from paid and unpaid work. Productivity costs will be calculated using the friction cost method. Absence will again be multiplied against standard cost prices [22]. QALYs will be calculated from utility scores from the SF-36 questionnaire, which will be administered at baseline, 1 month, 6 months, and 12 months [19,23]. Finally, costs and QALYs in both groups will be compared. It is our hypothesis that societal costs will be lower in the intervention group.

### Statistical Analyses

A power calculation was done using R software [24], which is based on a comparison of two proportions of patients with controlled BP (defined as an SBP of 139 mmHg or less and a DBP of 89 mmHg or less). We hypothesize that in the *The Box* group, 95% of the patients will achieve controlled BP, while in the control group, 75% will achieve controlled BP [25]. For this calculation, an alpha of 0.05, a beta of 0.20, and a margin of 0.07 were chosen, yielding a sample size of 200 patients.

Data will be analyzed according to the intention-to-treat principle. Causes of missing data will be tabulated (Multimedia Appendix 1). The percentage of missing data is expected to be low. Therefore, complete case analyses will be done. Analyses will be based on the missing-at-random assumption. If the percentage of missing values exceeds 7%, multiple imputation will be applied in the analysis of the data.

After finishing the study (defined as the last patient’s last visit), the proportion of patients with controlled BP will be compared with a Chi-square test. Logistic regression might be done if serious imbalances of baseline variables exist, to correct for potential confounding variables. Percentages of patients with controlled BP at three months (secondary outcome 1), and percentages of patients in which a previously unknown sustained arrhythmia is detected, will also be compared with a Chi-square test.

Scores of questionnaires (patient satisfaction questionnaire, MMAS-8 scale, iPAQ questionnaire, and SF-36 questionnaire) and health care utilization will be compared using an independent t-test. Major Adverse Cardiac Events (MACEs) will only be reported. As the study is underpowered, no statistics will be done on MACEs. The numbers will be hypothesis-generating.

## Discussion

In this paper, we presented the rationale and design of a single-center, open, RCT. With this trial, we will evaluate the clinical effectiveness of a smart technology intervention in patients with STEMI and NST-ACS.

### Clinical Effectiveness

It is expected that with daily monitoring of ECG, BP, weight, and steps, data will allow for early detection of high BP and the development of arrhythmias. For this study, the percentage of patients with controlled BP at 12 months has been chosen as primary outcome. Controlled BP is associated with lower risks of death, recurrent PCI, and stroke in patients who suffered from ACS. Therefore, the European guidelines recommend tight BP control via medication and lifestyle advice.

Percentages of 1-year mortality after STEMI or NST-ACS vary, but have been reported to be under 10% [26]. With a sample size of 200, it is expected that this study is underpowered to detect a significant difference in mortality between the intervention group and control group. It is emphasized that it is not the primary objective of this study to demonstrate a difference in mortality. This study is intended to investigate whether regular monitoring of clinical parameters (including BP) can lead to better control of those parameters, therefore improving surrogate outcomes in these patients.

### Patient Compliance

All devices used in the intervention group are designed for the consumer market. Patients will receive assistance with installation of the devices. After measurements, data will automatically be transferred to the hospital. It is expected that this automation will help to facilitate accurate and timely transmission, which might enhance patient compliance. To test this hypothesis, all reminders sent to patients for not having measured their data will be monitored. “No-shows” at digital outpatient clinic visits, and at the physical outpatient clinic visits, will be monitored as well. It is hypothesized that there will be no significant difference in the percentage of “no-shows” between the digital outpatient clinic visits and the physical outpatient clinic visits.

### Patient Satisfaction

During the study, patient satisfaction will be monitored via a validated questionnaire [18]. In this study, by design, patients in the intervention group will monitor ECG, BP, and weight more intensively. This increased frequency of monitoring has potential clinical benefits, such as having early detection of high BP or arrhythmias, as well as allowing patients to see and interpret their own data. This approach might enhance patient satisfaction of care. Conversely, daily monitoring might pose a burden to the patient, both physically (as patients must take time to perform the measurements) as well as mentally (as they might associate monitoring with their illness).

### Health Care Utilization

A concern regarding smart technology interventions is the fear that patients, given their nonmedical background and their perceived inability to interpreted medical data correctly, increase the number of contact points with hospitals, leading to more outpatient clinic visits and emergency department visits. This issue could therefore lead to higher burdens on both patients and health care staff, without improving clinical outcomes. Scientific evidence describing the relationship between increased monitoring frequency and health care utilization is scarce. Patients participating in this study will receive clear instructions about the usage of the devices, as well as the reasons for the hospital to contact the patients. It is therefore expected that health care utilization will not be higher in the intervention group.

### Generalizability

Patients with a STEMI or NST-ACS who match the inclusion and exclusion criteria will be eligible for participation. Patients who do not own a smartphone will not be excluded from the RCT, but patients who do not have Internet access at home will be excluded. This factor might affect generalizability. However, as 97% of the Dutch population has Internet access [27], it is expected that this exclusion criterion only slightly affects generalizability. The fact that a smartphone will be used for remote monitoring might affect generalizability as well. Previous literature has indicated that smartphone literacy decreases with age [28]. Furthermore, patients who do own a smartphone might refuse to participate as well, for various reasons [29]. It is also known that patients who participate in RCTs have different demographics than patients who do not [30]; it is therefore expected that generalizability will be affected. However, it is emphasized that this factor might partly be due to the involvement of smartphone technology and partly inherent to the RCT study design in general. As patients are given a smartphone in case they do not own one, and technical support is provided, generalizability issues will be kept to a minimum.

### Conclusion

In summary, the rationale and design of an RCT is presented to investigate whether a smart technology intervention can increase clinical effectiveness and patient satisfaction in the follow-up of STEMI or NST-ACS patients.

## Appendix

Multimedia Appendix 1 Example of how causes of missing data will be tabulated.

## Abbreviations

BP: blood pressure
CE: European Conformity
DBP: diastolic blood pressure
ECG: electrocardiogram
EMR: electronic medical record
FDA: Food and Drug Administration
iPAQ: International Physical Activity Questionnaire
LUMC: Leiden University Medical Center
MACE: Major Adverse Cardiac Event
MMAS: 8-item Morisky Medication Adherence Scale
NST-ACS: non-ST acute coronary syndrome
OS: operating system
PCI: percutaneous coronary intervention
PDF: portable document format
QALY: quality adjusted life year
RCT: randomized controlled trial
SBP: systolic blood pressure
SF-36: short form health questionnaire
STEMI: ST elevation myocardial infarction
TTE: transthoracic echocardiogram
