# Supplementary material for: Using Smart Technology to Improve Outcomes in Myocardial Infarction Patients: Rationale and Design of a Protocol for a Randomized Controlled Trial, The Box
Source: JMIR Res Protoc. 2017 Sep 22;6(9):e186. doi: 10.2196/resprot.8038 (PMC5630694; doi:10.2196/resprot.8038)
Supplement: Multimedia Appendix 1 [file resprot_v6i9e186_app1.pdf]

Appendix A

Supplement A

	Frequency of occurrence	Possible relationship with intervention (yes/no)
Cause I		
Cause II		
Cause III		
Cause IV		

Supplement A: example of how causes of missing data will be tabulated
